# Shotgun proteomics identification of proteins expressed in the Descemet’s membrane of patients with Fuchs endothelial corneal dystrophy

**DOI:** 10.1038/s41598-023-37104-1

**Published:** 2023-06-27

**Authors:** Tatsuya Nakagawa, Naoki Okumura, Masaya Ikegawa, Yumiko Toyama, Takashi Nirasawa, Frederic Mascarelli, Hanielle Vaitinadapoule, Ines Aouimeur, Zhiguo He, Philippe Gain, Gilles Thuret, Noriko Koizumi

**Affiliations:** 1 Department of Biomedical Engineering, Faculty of Life and Medical Sciences, Doshisha University, Kyotanabe, 610-0394 Japan; 2 Genomics, Proteomics and Biomedical Functions, Graduate School of Life and Medical Sciences, Doshisha University, Kyoto, Japan; 3 Bruker Japan K.K., Yokohama, Japan; 4 Laboratory of Biology, Engineering and Imaging for Ophthalmology (BiiO), Faculty of Medicine, Health Innovation Campus, Jean Monnet University, Saint-Étienne, France; 5 Centre de Recherche des Cordeliers, UMR S1138, Université Paris Descartes, Paris, France; 6 Department of Ophthalmology, University Hospital, Saint-Étienne, France

**Keywords:** Diseases, Eye diseases

## Abstract

Fuchs endothelial corneal dystrophy (FECD) is a slowly evolving, bilateral disease of the corneal endothelium, characterized by an abnormal accumulation of extracellular matrix (ECM) in the basement membrane (Descemet’s membrane, DM). This results in the formation of small round excrescences, called guttae, and a progressive disappearance of endothelial cells. In the intermediate stage, the numerous guttae create significant optical aberrations, and in the late stage, the loss of endothelial function leads to permanent corneal edema. The molecular components of guttae have not been fully elucidated. In the current study, we conducted shotgun proteomics of the DMs, including guttae, obtained from patients with FECD and revealed that 32 proteins were expressed only in the FECD-DMs but not in the DMs of control subjects. Subsequent enrichment analyses identified associations with multiple ECM-related pathways. Immunostaining of flat-mounted DMs confirmed that 4 of the top 5 identified proteins (hemoglobin α, SRPX2, tenascin-C, and hemoglobin γδεβ) were expressed in FECD-DMs but not in non-FECD-DMs. Fibrinogen α was strongly expressed in FECD-DMs, but weakly expressed in non-FECD-DMs. We also demonstrated that matrix-assisted laser desorption ionization imaging mass spectrometry (MALDI-IMS) can display the in situ spatial distribution of biomolecules expressed in the DM, including the guttae.

## Introduction

Fuchs endothelial corneal dystrophy (FECD) is a progressive, bilateral, and often inherited corneal endothelial disease^1–3^. The prevalence of FECD is approximately 4% over the age of 40 in the U.S.^4^, and 40% of corneal transplantations conducted worldwide are performed to treat FECD^5^. In the early stage, deposition of extracellular matrix (ECM) forms excrescences, called guttae, at the anterior chamber side of Descemet's membrane (DM)^6^. In the middle to advanced stage, the guttae increase, become confluent, and finally are partially covered with collagenous fibers associated with the loss of excrescence morphology^4,7^. During the progression of the disease, the corneal endothelium is continuously damaged, resulting in a decrease in cell density^1–4,7^.

Guttae are the clinical hallmark of FECD; indeed, the gold standard of FECD diagnosis is the identification of guttae by slit-lamp microscopy^8^. Guttae also induce high-order aberrations (HOAs) and light scattering, resulting in visual disturbance^9,10^. We previously proposed that the overproduction of ECM, which forms the guttae, induces corneal endothelial cell death by the unfolded protein response^11,12^. Despite the importance of guttae in vision and in the diagnosis and pathophysiology of FECD, many questions remain, such as the components of the guttae, the mechanism of formation of the excrescence morphology, and why guttae initiate at the corneal center and spread to the periphery. Guttae are composed of enormous molecules, but only some components, including fibronectin, type 1 collagen, type 4 collagen, type 8 collagen, laminin, and TGFBI, have been identified^13,14^. To our knowledge, a comprehensive analysis of the components of guttae has not yet been performed.

The progression of guttae in FECD is thought to involve pathological corneal endothelial cells, as endothelial cells are the only cells close to the DM and guttae. Therefore, one potential method for determining the components of guttae is transcriptome analysis of corneal endothelial cells. Chu and colleagues demonstrated the upregulation of multiple ECM-related genes and showed the activation of the fibrosis pathway by conducting an Ingenuity Pathway Analysis (IPA)^15^. Consistently, we found that ECM molecules, such as biglycan (*BGN*), chitinase 3 like 1 (*CHI3L1*), collagen type VI alpha 2 chain (*COL6A2*), fibronectin 1 (*FN1*), and matrilin 3 (*MATN3*), showed significantly higher expression in the corneal endothelial cells of patients with FECD than in non-FECD control subjects^16^. However, the expression levels of mRNA and protein do not always correlate, due to post-transcriptional regulation and alternative splicing. In addition, upregulated ECM genes in corneal endothelial cells only indirectly suggest that these molecules are potential components of guttae.

In the current study, we conducted shotgun proteomics of the DM, including guttae, to obtain a comprehensive identification of proteins upregulated in FECD. A further validation study was performed by immunofluorescence staining of DMs obtained from patients with FECD. We also evaluated the feasibility of using matrix-assisted laser desorption ionization imaging mass spectrometry (MALDI-IMS) to characterize the in situ spatial distribution of biomolecules in the DM.

## Results

### Shotgun proteomics of Descemet's membranes from patients with FECD and non-FECD controls

**Figure 1:**
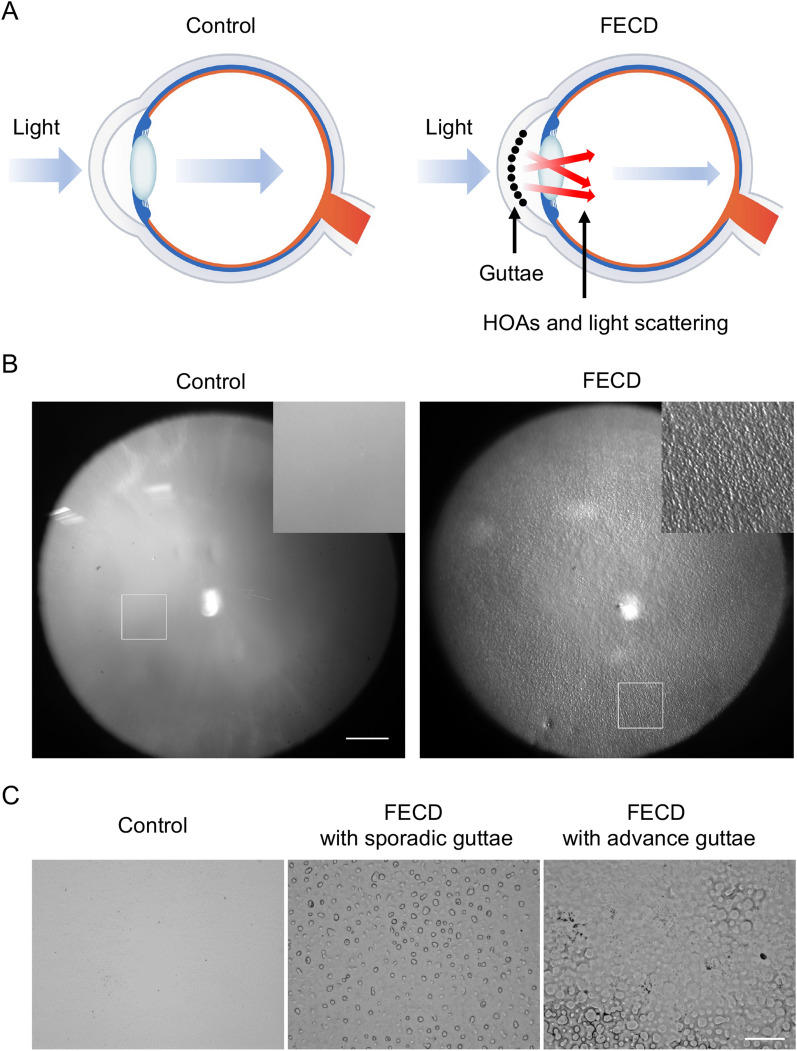
Guttae on Descemet's membrane (DM) of a subject with FECD (Fuchs endothelial corneal dystrophy). (**A**) In FECD, guttae reduce vision due to increased high-order aberrations (HOAs) and light scattering. (**B**) Representative retrocorneal illumination image obtained by modified slit-lamp microscopy showed confluent guttae at the corneal center, surrounded by a less confluent area at the mid-periphery in patients with FECD. By contrast, no guttae are observed in the non-FECD control subject. Insert shows a × 3 magnified image of the regions of interest. Scale bar: 1 mm. (**C**) Flat-mounted Descemet’s membrane (DM) showed a homogenous sheet without guttae in a non-FECD subject (left). By contrast, guttae were observed in a sporadic pattern with excrescence morphology in the subject with FECD (middle). In the advanced stage of FECD, the guttae became larger and fused (right). Scale bar: 200 μm.

Guttae reduce vision due to increases in HOAs and light scattering^9,10^, and they are important clinical findings that are used by physicians to decide the timing of surgical intervention (Fig. 1A). Our representative retrocorneal illumination images obtained by a modified ophthalmology slit-lamp microscope showed that the guttae in patients with FECD exhibit a confluent area at the corneal center, surrounded by a less confluent area at the mid-periphery (Fig. 1B). Insertion of the magnified image of the mid-periphery area revealed the morphology of the excrescences. By contrast, no guttae were observed in the non-FECD control subject. Likewise, flat-mounted DMs showed that guttae exhibited sporadic pattern with excrescence morphology in the FECD subject (Fig. 1C, middle), but the DM was homogenous, without guttae, in the non-FECD subject (Fig. 1C, left). In the advanced stage of FECD, the guttae became larger and fused, and individual guttae became difficult to distinguish (Fig. 1C, right).

**Figure 2:**
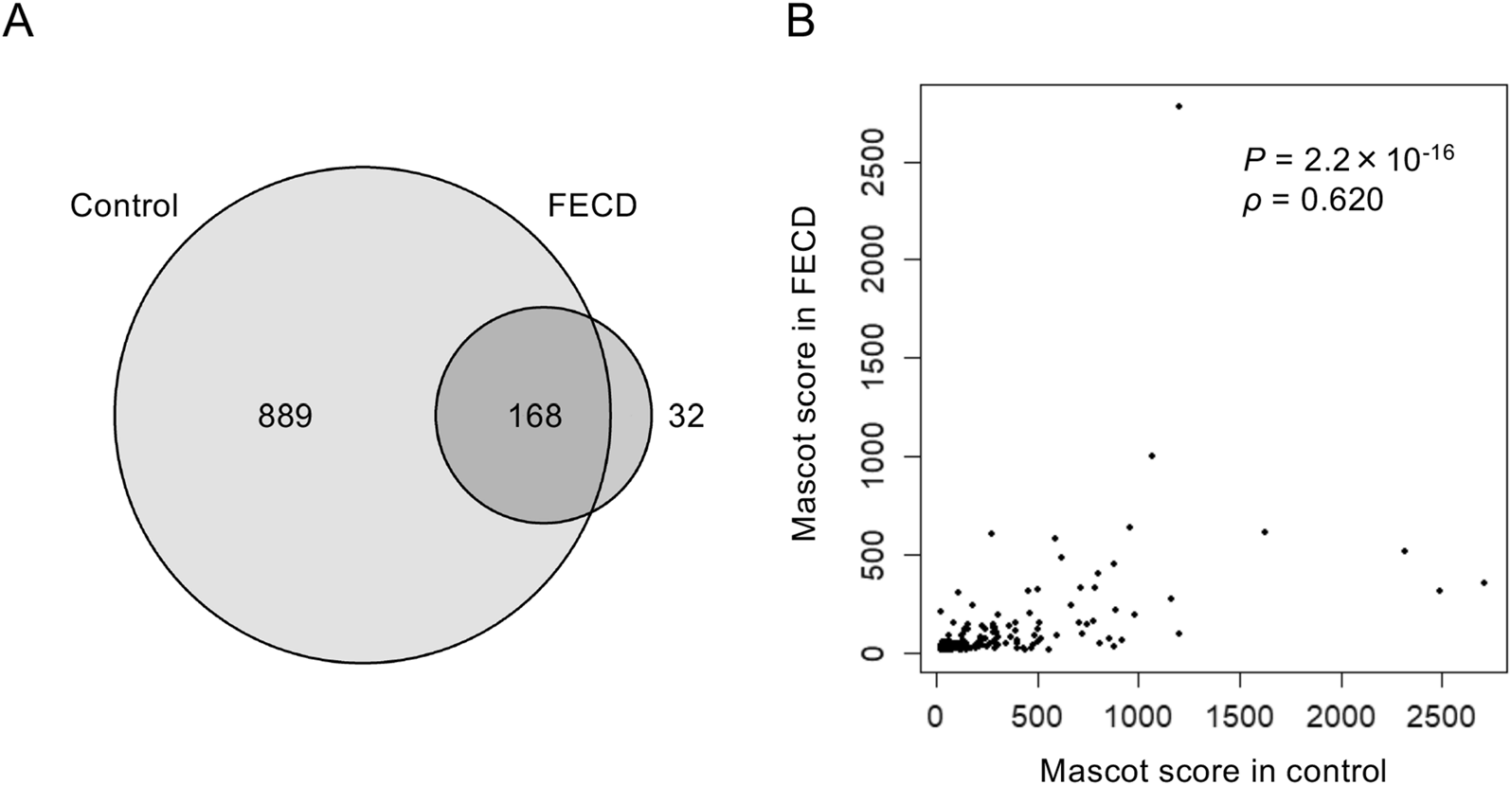
Shotgun proteomics of Descemet's membrane (DM) of non-FECD (Fuchs endothelial corneal dystrophy) and FECD subjects. (**A**) Proteins of DM of non-FECD and FECD subjects without the presence of corneal endothelium were analyzed by shotgun proteomics. The 1057 proteins in the non-FECD-DM and 200 proteins in the FECD-DM were identified. Venn diagram shows 168 proteins were commonly identified in the DMs of subjects with and without FECD, while 32 proteins were identified only in the DM with FECD and 889 proteins were identified only in the DM without FECD. (**B**) Spearman's rank correlation coefficient showed a positive correlation between the Mascot scores of non-FECD-DM and FECD-DM (ρ = 0.620, *P*-value = 2.2 × 10^–16^). The correlation was mild, and some scatter plots were dislocated from the linear correlation.

Shotgun proteomics identified 1057 proteins in the DM of the non-FECD subject (Sample ID C7 in Table 1) and 200 proteins in the DM of the FECD-DM subject (Sample ID F7 in Table 1). A Venn diagram showed that 168 proteins were commonly expressed in both the non-FECD and FECD samples, while 32 proteins were expressed only in the FECD-DM and 889 proteins were expressed only in the non-FECD-DM (Fig. 2A). The Spearman's rank correlation coefficient indicated a positive correlation between the Mascot scores of the non-FECD control and FECD samples (ρ = 0.620, *P*-value = 2.2 × 10^–16^). However, the correlation was mild, and some scatter plots were dislocated from the linear correlation (Fig. 2B).

**Table 1:** Sample information.

Sample ID	Category	Age (years)	Sex	Post-mortem time (hours)	Applications
C1	Non-FECD	87	Female	20	Preliminary experiments for shotgun analysis
C2	Non-FECD	87	Female	20	Preliminary experiments for shotgun analysis
C3	Non-FECD	72	Female	19	Preliminary experiments for MALDI-IMS
C4	Non-FECD	74	Male	24	Preliminary experiments for MALDI-IMS
C5	Non-FECD	52	Female	22	Preliminary experiments for MALDI-IMS
C6	Non-FECD	74	Male	24	Preliminary experiments for MALDI-IMS
C7	Non-FECD	80	Male	9	MALDI-IMS and Shotgun analysis
C8	Non-FECD	77	Male	7	Immunostaining
C9	Non-FECD	104	Female	24	Immunostaining
C10	Non-FECD	76	Female	13	Immunostaining
F1	FECD	72	Male	–	Preliminary experiments for shotgun analysis
F2	FECD	84	Female	–	Preliminary experiments for shotgun analysis
F3	FECD	58	Male	–	Preliminary experiments for MALDI-IMS
F4	FECD	44	Male	–	Preliminary experiments for MALDI-IMS
F5	FECD	81	Female	–	Preliminary experiments for MALDI-IMS
F6	FECD	66	Male	–	Preliminary experiments for MALDI-IMS
F7	FECD	63	Female	–	MALDI-IMS and Shotgun analysis
F8	FECD	58	Female	–	Immunostaining
F9	FECD	77	Female	–	Immunostaining
F10	FECD	90	Female	–	Immunostaining

The top 30 proteins expressed in DM of the non-FECD subject and the patient with FECD are shown in Tables 2 and 3, respectively. Overall, 32 proteins were identified only in the FECD-DM and not in the non-FECD-DM (Table 4). Eleven of the top 32 proteins (biglycan, collagen type VI alpha 2 chain, collagen type VIII alpha 1 chain, collagen type XVIII alpha 1 chain, latent transforming growth factor beta binding protein 2, lumican, matrilin 2, matrilin 3, mucin 6, oligomeric mucus/gel-forming, proline and arginine rich end leucine rich repeat protein, and tenascin C) were expressed only in the FECD-DM and are characterized as ECM molecules according to their Gene Ontology (GO) annotation (Category ID: GO:0031012).

**Table 2:** Top 30 proteins identified in the Descemet’s membrane of a control subject without Fuchs endothelial corneal dystrophy.

Protein name	Gene symbol	Ensembl gene ID	UniProtKB ID	Mascot score
Myosin-9	*MYH9*	ENSG00000100345	P35579	2709.9
Plectin	*PLEC*	ENSG00000178209	Q15149	2487.9
Myosin-10	*MYH10*	ENSG00000133026	P35580	2312.7
Vimentin	*VIM*	ENSG00000026025	P08670	1621.2
Neuroblast Differentiation-Associated Protein AHNAK	*AHNAK*	ENSG00000124942	Q09666	1465.6
Endoplasmic reticulum chaperone BiP	*HSPA5*	ENSG00000044574	P11021	1200.7
Collagen alpha-1(XII) chain	*COL12A1*	ENSG00000111799	Q99715	1197.3
Alpha-enolase	*ENO1*	ENSG00000074800	P06733	1158.9
Transforming growth factor-beta-induced protein ig-h3	*TGFBI*	ENSG00000120708	Q15582	1066.3
Endoplasmin	*HSP90B1*	ENSG00000166598	P14625	980.3
Basement membrane-specific heparan sulfate proteoglycan core protein	*HSPG2*	ENSG00000142798	P98160	957.3
Protein disulfide-isomerase A3	*PDIA3*	ENSG00000167004	P30101	914.8
ATP synthase subunit beta, mitochondrial	*ATP5B*	ENSG00000110955	P06576	886.5
Laminin subunit alpha-5	*LAMA5*	ENSG00000130702	O15230	877.7
Myocilin	*MYOC*	ENSG00000034971	Q99972	876.9
Pyruvate kinase PKM	*PKM*	ENSG00000067225	P14618	856.7
60 kDa heat shock protein, mitochondrial	*HSPD1*	ENSG00000144381	P10809	810.6
ATP synthase subunit alpha, mitochondrial	*ATP5A1*	ENSG00000152234	P25705	797.8
Actin, cytoplasmic 1	*ACTB*	ENSG00000075624	P60709	785.6
Ribosome-binding protein 1	*RRBP1*	ENSG00000125844	Q9P2E9	776.2
Glyceraldehyde-3-phosphate dehydrogenase	*GAPDH*	ENSG00000111640	P04406	775.3
Prelamin-A/C	*LMNA*	ENSG00000160789	P02545	743.4
Heat shock protein HSP 90-beta	*HSP90AB1*	ENSG00000096384	P08238	723.6
Annexin A2	*ANXA2*	ENSG00000182718	P07355	711.7
Stress-70 protein, mitochondrial	*HSPA9*	ENSG00000113013	P38646	709.5
Cytoskeleton-associated protein 4	*CKAP4*	ENSG00000136026	Q07065	668.1
Heat shock cognate 71 kDa protein	*HSPA8*	ENSG00000109971	P11142	645.5
Sodium/potassium-transporting ATPase subunit alpha-1	*ATP1A1*	ENSG00000163399	P05023	620.0
Fructose-bisphosphate aldolase A	*ALDOA*	ENSG00000149925	P04075	596.2

**Table 3:** Top 30 proteins identified in the Descemet’s membrane of a patient with Fuchs endothelial corneal dystrophy.

Protein name	Gene symbol	Ensembl gene ID	UniProtKB ID	Mascot score
Collagen alpha-1(XII) chain	*COL12A1*	ENSG00000111799	Q99715	2781.1
Transforming growth factor-beta-induced protein ig-h3	*TGFBI*	ENSG00000120708	Q15582	1003.6
Basement membrane-specific heparan sulfate proteoglycan core protein	*HSPG2*	ENSG00000142798	P98160	633.9
Vimentin	*VIM*	ENSG00000026025	P08670	613.5
Collagen alpha-3(VI) chain	*COL6A3*	ENSG00000163359	P12111	602.5
Thrombospondin-1	*THBS1*	ENSG00000137801	P07996	577.8
Myosin-10	*MYH10*	ENSG00000133026	P35580	510.7
Sodium/potassium-transporting ATPase subunit alpha-1	*ATP1A1*	ENSG00000163399	P05023	483.3
Laminin subunit alpha-5	*LAMA5*	ENSG00000130702	O15230	452.0
ATP synthase subunit alpha, mitochondrial	*ATP5A1*	ENSG00000152234	P25705	399.1
Myosin-9	*MYH9*	ENSG00000100345	P35579	355.3
Annexin A2	*ANXA2*	ENSG00000182718	P07355	327.1
Actin, cytoplasmic 1	*ACTB*	ENSG00000075624	P60709	326.4
EMILIN-1	*EMILIN1*	ENSG00000138080	Q9Y6C2	316.2
Clusterin	*CLU*	ENSG00000120885	P10909	310.2
Laminin subunit gamma-1	*LAMC1*	ENSG00000135862	P11047	308.4
Plectin	*PLEC*	ENSG00000178209	Q15149	308.3
Fibronectin	*FN1*	ENSG00000115414	P02751	303.8
Alpha-enolase	*ENO1*	ENSG00000074800	P06733	268.5
Histone H4	*HIST1H4I*	ENSG00000276180	P62805	239.7
Fibrinogen alpha chain	*FGA*	ENSG00000171560	P02671	238.9
Cytoskeleton-associated protein 4	*CKAP4*	ENSG00000136026	Q07065	237.8
ATP synthase subunit beta, mitochondrial	*ATP5B*	ENSG00000110955	P06576	218.3
Hemoglobin subunit alpha	*hba1*	ENSG00000206172	P69905	208.3
Hemoglobin subunit beta	*HBB*	ENSG00000244734	P68871	205.7
Filamin-A	*FLNA*	ENSG00000196924	P21333	197.5
Annexin A1	*ANXA1*	ENSG00000135046	P04083	193.1
Endoplasmin	*HSP90B1*	ENSG00000166598	P14625	190.4
Glyceraldehyde-3-phosphate dehydrogenase	*GAPDH*	ENSG00000111640	P04406	159.2

**Table 4:** Thirty-two proteins identified only in the Descemet’s membrane of patients with Fuchs endothelial corneal dystrophy (FECD) but not in the DM of control non-FECD subjects.

Protein name	Gene symbol	Ensembl gene ID	UniProtKB ID	Mascot score
Fibrinogen alpha chain	*FGA*	ENSG00000171560	P02671	238.9
Hemoglobin subunit alpha	*HBA1*	ENSG00000206172	P69905	208.3
Sushi repeat-containing protein SRPX2	*SRPX2*	ENSG00000102359	O60687	154.8
Tenascin	*TNC*	ENSG00000041982	P24821	142.6
Hemoglobin subunit delta	*HBD*	ENSG00000223609	P02042	115.8
Keratin, type II cytoskeletal 7	*KRT7*	ENSG00000135480	P08729	115.4
Tubulin alpha-1A chain	*TUBA1A*	ENSG00000167552	Q71U36	109.3
Histone H2B type 1-K	*Hist1h2bk*	ENSG00000197903	O60814	96.0
Keratin, type II cytoskeletal 1b	*KRT77*	ENSG00000189182	Q7Z794	91.3
Matrilin-2	*MATN2*	ENSG00000132561	O00339	71.4
Collagen alpha-1 (XVIII) chain	*COL18A1*	ENSG00000182871	P39060	70.6
Prolargin	*PRELP*	ENSG00000188783	P51888	60.7
Collagen alpha-2 (VI) chain	*COL6A2*	ENSG00000142173	P12110	57.5
Collagen alpha-1 (VIII) chain	*COL8A1*	ENSG00000144810	P27658	51.2
Lumican	*LUM*	ENSG00000139329	P51884	50.9
Matrilin-3	*MATN3*	ENSG00000132031	O15232	50.6
Fibrinogen beta chain	*FGB*	ENSG00000171564	P02675	44.7
Fibrinogen gamma chain	*FGG*	ENSG00000171557	P02679	37.8
Adipocyte enhancer-binding protein 1	*AEBP1*	ENSG00000106624	Q8IUX7	32.7
Biglycan	*BGN*	ENSG00000182492	P21810	31.8
Solute carrier family 2, facilitated glucose transporter member 3	*SLC2A3*	ENSG00000059804	P11169	28.8
Sodium/potassium-transporting ATPase subunit beta-1	*ATP1B1*	ENSG00000143153	P05026	26.6
HLA class I histocompatibility antigen, alpha chain F	*HLA-F*	ENSG00000204642	P30511	24.9
Protein FAM162A	*FAM162A*	ENSG00000114023	Q96A26	23.9
Latent-transforming growth factor beta-binding protein 2	*LTBP2*	ENSG00000119681	Q14767	21.7
LEM domain-containing protein 2	*LEMD2*	ENSG00000161904	Q8NC56	20.9
Angiopoietin-related protein 7	*ANGPTL7*	ENSG00000171819	O43827	18.8
Sodium/nucleoside cotransporter 1	*SLC28A1*	ENSG00000156222	O00337	15.7
EMILIN-2	*EMILIN2*	ENSG00000132205	Q9BXX0	15.2
Pyruvate dehydrogenase E1 component subunit alpha, testis-specific form, mitochondrial	*PDHA2*	ENSG00000163114	P29803	15.0
Mucin-6	*MUC6*	ENSG00000184956	Q6W4X9	14.5
26S proteasome regulatory subunit 6A	*PSMC3*	ENSG00000165916	P17980	14.2

### Enrichment analyses of proteins expressed in the DM

**Figure 3:**
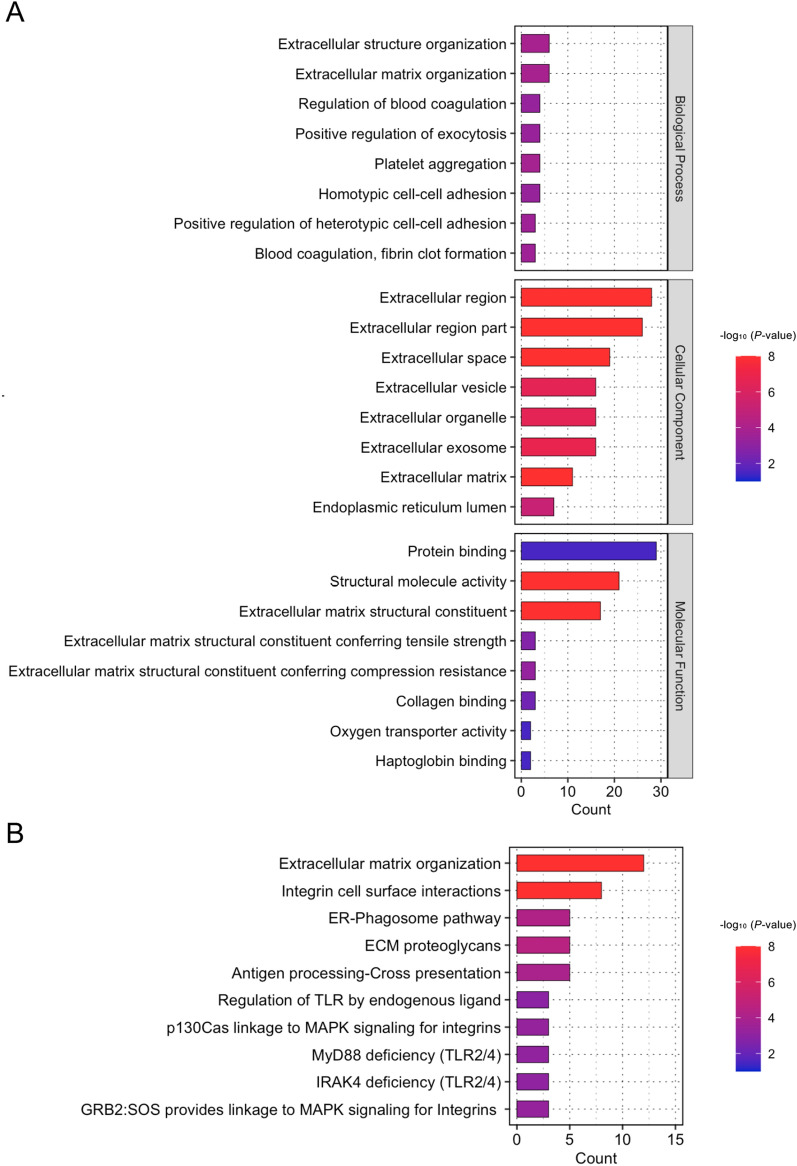
Gene ontology and reactome analyses of 32 proteins identified only in Descemet’s membrane (DM) of patients with FECD (Fuchs endothelial corneal dystrophy). (**A**) The 32 proteins identified only in the DM of the patient with FECD but not in the DM of non-FECD subject were subjected to the Gene Ontology (GO) analysis. The top 8 significantly enriched GO terms of the biological process, cellular component, and molecular function are shown. Multiple extracellular matrix-related GO terms were enriched. The significance of each GO term was represented by -log10 (*P*-value) with a color bar ranging from blue to red. (**B**) The 32 proteins identified only in the DM of the patient with FECD were subjected to the Reactome analysis. Multiple extracellular matrix-related pathways involving ECM organization, integrin cell surface interaction, and ECM proteoglycans were significantly enriched. The color bar indicates the significance of each pathway represented by − log10 (*P*-value) ranging from blue to red.

The 32 proteins expressed only in the DM of the patient with FECD were subjected to Gene Ontology (GO) analysis. The top 8 significantly enriched GO terms of biological process, cellular component, and molecular function are shown in Fig. 3A. The significance of each GO term was represented by -log10 (*P*-value) and a color bar ranging from blue to red. All GO terms of biological process, cellular component, and molecular function were significantly enriched in multiple ECM-related pathways. For instance, the extracellular matrix (GO:0031012) included 11 proteins (biglycan, collagen type VI alpha 2 chain, collagen type VIII alpha 1 chain, collagen type XVIII alpha 1 chain, latent transforming growth factor beta binding protein 2, lumican, matrilin 2, matrilin 3, mucin 6 oligomeric mucus/gel-forming, proline and arginine rich end leucine rich repeat protein, and tenascin C) for the GO term of cellular component. Likewise, reactome pathway analysis showed enrichment of multiple ECM-related pathways in the FECD-DM (Fig. 3B). These enriched ECM-associated pathways revealed by GO and reactome pathway analyses were consistent with the clinical findings that patients with FECD exhibited guttae formation and thickened DM composed mainly of extracellular matrix. This finding supported the feasibility of using shotgun proteomics to identify the proteins expressed in the DM.

### Immunostaining of the DMs obtained from subjects with and without FECD

**Figure 4:**
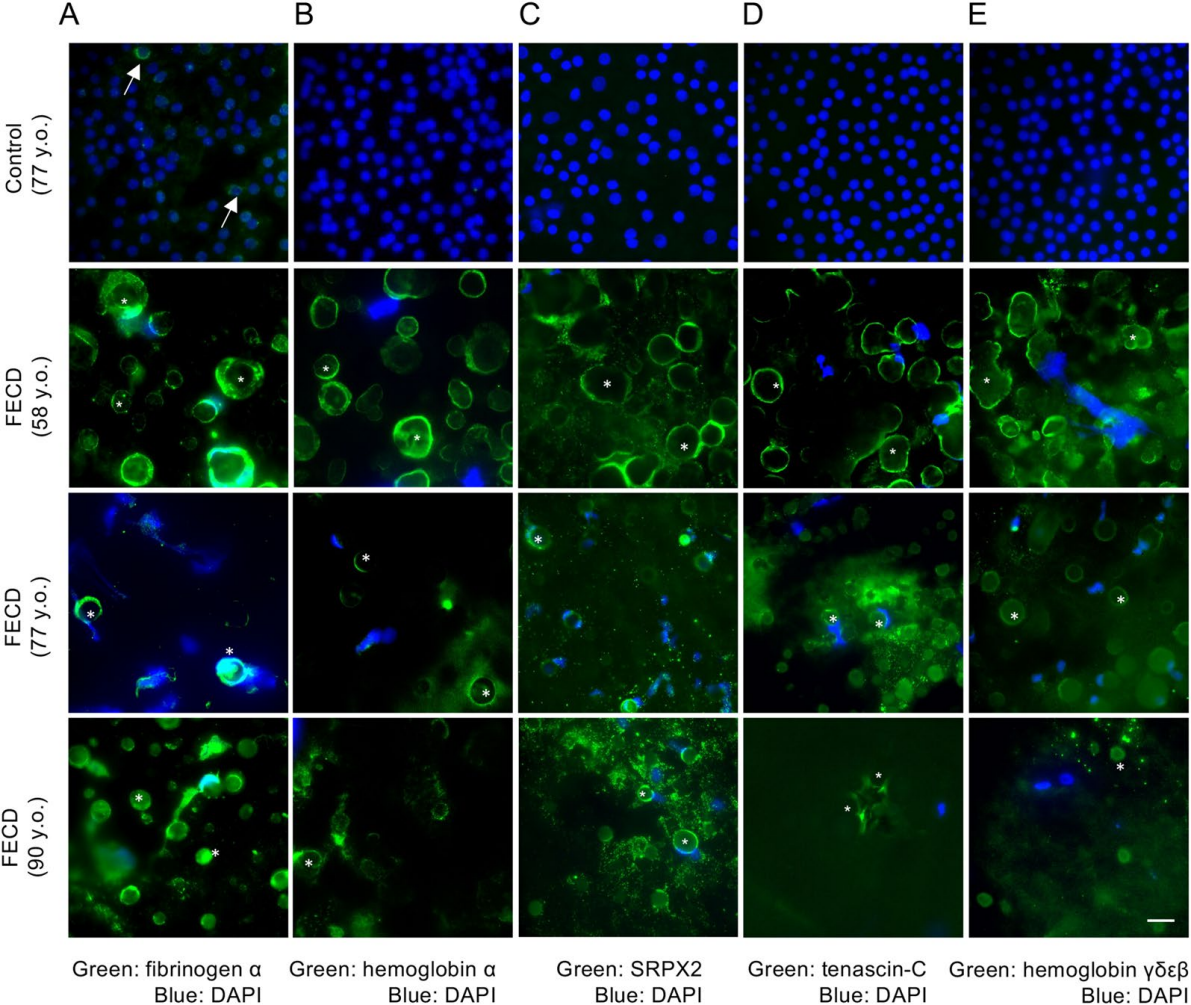
Immunostaining of flat mounts of Descemet's membranes (DMs) obtained from subjects with and without FECD (Fuchs endothelial corneal dystrophy). (**A**) In the control sample, weak perinuclear staining of the fibrinogen α chain was sporadically observed in the non-FECD-DM. Arrows indicate the perinuclear expression of the fibrinogen α chain. In the FECD-DMs, the fibrinogen α chain was clearly stained at the outer surface of the guttae. Asterisks indicate guttae in the FECD-DM. (**B**) In the non-FECD-DM, no staining was observed. Hemoglobin subunit α showed staining at the outer surface of the guttae in the FECD-DMs. (**C**) In the non-FECD samples, no staining of sushi repeat containing protein x-linked 2 (SRPX2) was observed. In the FECD-DMs, SRPX2 was stained at the surface of the guttae and on the DM close to the guttae. (**D**) In the non-FECD-DM, no staining of tenascin-C was observed. In the FECD-DMs, tenascin-C was stained at the surface of the guttae, and the dot-like staining pattern was also observed on the DM close to the guttae. (**E**) In the non-FECD-DM, no staining of hemoglobin subunit γδεβ was observed. In the FECD-DMs, hemoglobin subunit γδεβ was stained at the surface of the guttae and on the DM close to the guttae. The nuclei were counterstained with DAPI. Immunostaining of each protein was repeated in the DM obtained from 3 patients with FECD and 3 non-FECD (control) donors, and images of 3 patients with FECD and representative images of the control are shown. The scale bars correspond to 20 µm.

We evaluated the expression of the top 5 of the 32 proteins expressed only in the FECD-DM but not in the non-FECD control by immunofluorescence staining to validate the proteins identified by shotgun proteomics (Fig. 4 and Supplementary Fig. 1). DMs with corneal endothelium were used to evaluate the distribution of the proteins for immunostaining. In the non-FECD control samples, only fibrinogen α showed weak staining, while the other 4 proteins (hemoglobin α, SRPX2, tenascin-C, and hemoglobin γδεβ) were not detected. In the FECD-DM, fibrinogen α, hemoglobin α, SRPX2, tenascin-C, and hemoglobin γδεβ were clearly detected by immunostaining. Fibrinogen α and hemoglobin α were mainly associated with guttae, whereas SPX2, tenascin-C, and hemoglobin γδεβ were detected in the guttae and on the surface of the DM close to the guttae. Due to the corneal endothelial cell damage induced by FECD and the unavoidable mechanical trauma occurring during descemetorhexis, only a limited number of cells were observed in the FECD samples. By contrast, the non-FECD control samples obtained from donor corneas showed an almost confluent cell layer. A negative control using rabbit and mouse non-specific IgGs as the primary antibodies showed no specific staining of the DMs from either the non-FECD control or FECD samples (data not shown). In total, 3 non-FECD and 3 FECD samples were evaluated, and representative images are shown in Fig. 4.

### Feasibility of MALDI-IMS of the DM

**Figure 5:**
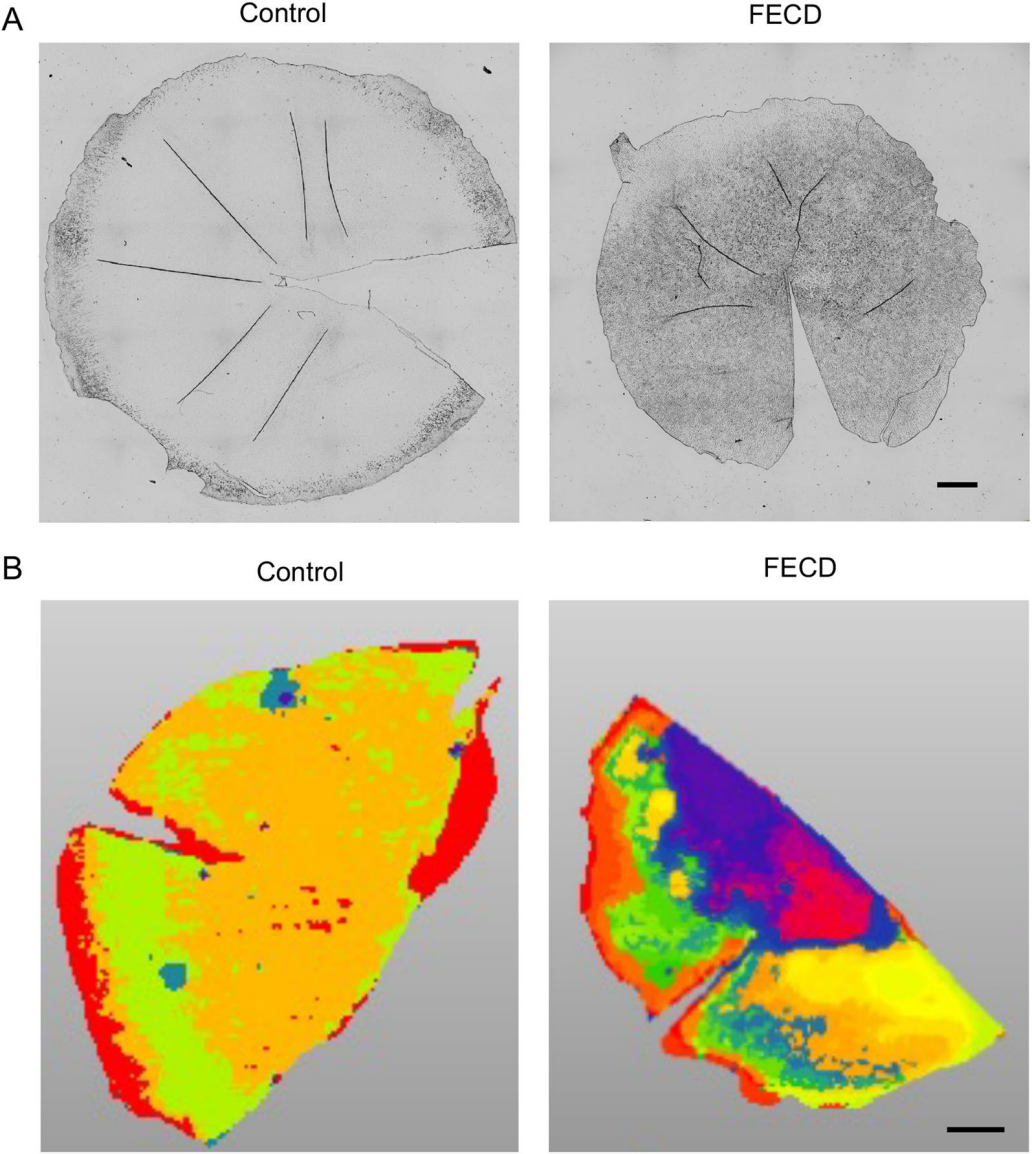
Matrix-assisted laser desorption ionization imaging mass spectrometry (MALDI-IMS) of Descemet's membrane (DM) of non-FECD (Fuchs endothelial corneal dystrophy) and FECD subjects. (**A**) Flat-mount images of the DMs obtained from non-FECD donor corneas and patient with FECD are shown. The non-FECD-DM showed a homogenous sheet without guttae, but with a presumed curly structure in the very peripheral area. The FECD-DM showed massive guttae throughout the whole area, while higher confluency was observed in the central part than in the periphery. Scale bar: 1 mm. (**B**) MALDI-IMS of non-FECD-DM displayed mainly yellow and green colors. By contrast, the FECD-DM displayed purple and red areas, presumably reflecting the confluent guttae at the paracentral area surrounded by yellow and green areas. Representative images of three independent experiments in each group are shown. Scale bar: 1 mm.

A representative microscopic image of flat-mounted DM obtained from a non-FECD donor cornea (Sample ID C4 shown in Table 1) showed a homogenous sheet without guttae but a presumed so-called curly structure in the most peripheral area^17^. By contrast, DM derived from a patient with FECD (Sample ID F4 shown in Table 1) showed massive guttae throughout almost all the 8 mm diameter, with a confluent guttae area in the center (Fig. 5A). (Note that the diameter of the FECD-DM obtained during corneal endothelial keratoplasty was approximately 8 mm, while that of the non-FECD-DM control from the donor cornea was approximately 12 mm.) The flat-mounted DMs were cut into 2 pieces, with one used for MALDI-IMS and the other for shotgun proteomics. The acquired image data were investigated using unsupervised multivariate statistics to obtain image segmentation of the anatomical regions of interest based on their chemical identities. The image segmentation identified by probabilistic latent semantic analysis (pLSA) of the non-FECD-DM control (Sample ID C7 shown in Table 1) displayed mainly yellow and green colors, reflecting the expression of specific molecules. By contrast, the MALDI-IMS of the FECD-DM sample (Sample ID F7 shown in Table 1) displayed purple and red areas in the center (slightly dislocated), presumably reflecting the confluent guttae, and yellow and green areas in the mid-periphery. These results verified that the MALDI-IMS could be utilized for in situ visualization of the expressed molecules in flat-mounted DMs (Fig. 5B).

## Discussion

Guttae are usually observed in the bilateral eyes by slit-lamp microscopy in the clinical setting and have been pivotal findings for diagnosing FECD^8^. In addition to the importance of guttae as a diagnostic finding, guttae reduce vision. Impaired vision in patients with FECD is induced for two reasons: (1) an increase in HOAs and light scattering due to the presence of guttae in the intermediate stage of the disease and (2) loss of corneal transparency due to endothelial decompensation, which causes permanent stromal edema in the advanced stage^2,3^. For many years, corneal transplantation, consisting of replacement with a full-thickness cornea (penetrating keratoplasty), was the only therapy for FECD. A loss of corneal transparency induced by a drop in cell density to a certain threshold was the main indication for transplantation. In the last decade, endothelial transplantations, such as Descemet’s stripping automated endothelial keratoplasty (DSAEK) and Descemet’s membrane endothelial keratoplasty (DMEK), have been introduced and are now the first-line therapy^18–20^. Endothelial transplantations are less invasive and faster and provide better visual recovery than conventional penetrating keratoplasty; consequently, the number of endothelial transplantations has rapidly increased, and indications have become wider to now allow treatment of the earlier stages of FECD^18–21^. Indeed, visual disturbance presumably due to guttae in patients maintaining a non-edematous cornea is currently accepted as an indication for endothelial transplantation. In addition, the surgical procedure of Descemet’s membrane stripping only (DSO), in which guttae are removed by stripping the central DM together with the guttae, has been proposed as an effective treatment for early-stage FECD^22–28^, although larger analyses with long-term follow-ups are still necessary^29^. Visual recovery following DSO implicates guttae as the cause of FECD-induced visual disturbance.

Many clinical studies now support the notion that guttae impair vision in eyes without corneal edema. For instance, Watanabe and colleagues reported that guttae induced intraocular forward light scattering, and they proposed that the area affected by guttae was correlated with visual acuity, contrast sensitivity, and stray light^10^. Wacker and colleagues reported that anterior and posterior HOAs and backscatter are higher in FECD eyes than in normal eyes, even in the early stage of FECD without corneal edema^9^. In addition, accumulating evidence shows the dynamic reciprocity between guttae and corneal endothelial cells in patients with FECD, indicating the important role of guttae in FECD pathogenesis^30,31^. These current clinical and research trends highlight the importance of guttae, and this motivated us to investigate the guttae and DM of patients with FECD using modern research methodologies.

In this study, shotgun proteomics revealed 32 proteins that were expressed only in the FECD-DM and not in the non-FECD-DM. Eleven of the 32 proteins are ECM molecules, suggesting that those proteins are potentially responsible for the accumulation of pathological molecules composing guttae and the collagenous fibers that cover the guttae in the advanced stage. To validate our shotgun proteomics results, we conducted immunostaining for the top 5 of the 32 proteins that were identified by proteomics as proteins only expressed in the FECD-DM. Four proteins were stained only in FECD samples, while fibrinogen α showed weak staining in non-FECD-DM (but was clearly stained in the FECD-DM). This weak immunostaining of fibrinogen α suggests the possibility of different detection sensitivity between immunostaining and shotgun analysis, suggesting that validation by different methodologies is necessary. Our current shotgun proteomics identified 1057 proteins in the DM of non-FECD subject and 200 proteins in the FECD-DM. One potential explanation for the discrepancies in the numbers of identified proteins might be mostly due to the structural change in FECD-DM, resulting in hampering the effective protein extraction. As we established the pipeline for analyzing flat-mounted DM by shotgun proteomics, we are currently collecting in-depth proteomics data from a larger number of samples to compensate above mentioned limitation. In future experiments, the integration of shotgun proteomics and mass spectrometry imaging will add novel findings for elucidating corneal pathology.

We recently conducted RNA-seq of corneal endothelial cells from patients with FECD and from non-FECD subjects, and we identified 2366 differentially expressed genes (1092 upregulated and 1274 downregulated genes)^16^. Of the 32 proteins expressed only in the FECD sample in the current study, our previous RNA-seq analysis revealed that 10 genes, coding for tenascin C, sushi repeat-containing protein SRPX2, adipocyte enhancer-binding protein 1, latent-transforming growth factor beta-binding protein 2, matrilin-3, keratin type II cytoskeletal 7, collagen alpha-2(VI) chain, collagen alpha-1(VIII) chain, and biglycan, hemoglobin subunit alpha 1, were upregulated at the transcriptional level. The identification of the remaining 22 proteins only in the FECD sample in this study and not as upregulated genes in previous RNA-seq analysis suggests the possibility of impaired degradation of pathological proteins in FECD, although further studies are necessary.

The FECD-related guttae appear in a heterogeneous morphological pattern with multiple molecular components^4,7^; therefore, understanding the spatial distribution of these molecules is important for understanding the mechanism of guttae formation. In the present study, our motivation was to utilize MALDI-IMS to display the multiple molecule expressions in pathological DM, including in the guttae. MALDI-IMS is an in situ label-free visualization method that combines mass spectrometry and molecular imaging^32–35^. Its rapid progress in the last decade has led to increased sensitivity, reduced acquisition time, and higher spatial resolution. MALDI-IMS has been utilized in the study of several diseases to elucidate the pathophysiology and biomarkers and to identify novel therapeutic targets^34^. However, identifying the detected molecules in MALDI-IMS is challenging and results in the extraction of insufficient information^36^. Therefore, the complementation of MALDI-IMS with orthogonal shotgun proteomics has been investigated. For instance, a desktop application, ImShot, was recently developed to automatically integrate the data from MALDI-IMS and shotgun proteomics^36^. The spatial resolution of the currently utilized MALDI-IMS was 50 μm, suggesting that guttae with diameter ranges of 20–400 μm^31,37,38^ can be detected. However, discriminating a single gutta from the specimen and identifying its expressed proteins may require other approaches, such as laser-capture microdissection^39,40^.

One potential drawback of this study is the lack of genetic information on patients with FECD. Trinucleotide repeat expansion in TCF4 is the most frequent genetic abnormality reported in patients with FECD, and it is proposed to induce multiple pathophysiological pathways^41^. The effect of repeat expansion on protein expression is worth investigating further using current metrologies, including MALDI-IMS and shotgun proteomics, as the results might prove beneficial for understanding the role of trinucleotide repeat expansions in the formation of guttae.

In the present study, we showed the feasibility of our pipeline by dividing the DM from a single patient into 2 pieces and subjecting one to MALDI-IMS and the other to shotgun proteomics. Future studies aimed at analyzing DM samples at various disease stages by integrating the data from MALDI-IMS and shotgun proteomics will be beneficial for understanding FECD pathophysiology and identifying FECD biomarkers. Our study also showed that flat-mounted DMs, without paraffin embedding or sectioning, can be studied directly with MALDI-IMS. The current methodology using the combination of MALDI-IMS and shotgun proteomics could prove to be a powerful new research tool.

## Methods

### Ethical approval

This study was approved by the Institutional Review Board of the Doshisha University (No. 20032), the Jean Monnet University (No Siret: 194 210 951 00423, Code: NAF/APE. 8542Z), and the University Hospital of Saint-Etienne (No Siret: 26420030400055, Code NAF/APE: 8610Z). This study was conducted in accordance with the ethical principles of the Declaration of Helsinki. Written informed consent was obtained from all patients with FECD. All DM samples were obtained at the University Hospital Center of Saint Etienne. The non-FECD human donor corneas were procured at the laboratory of anatomy (Body Donation to Science) of the Faculty of Medicine of Jean Monnet University. No tissues were procured from prisoners.

### Acquisition of the retrocorneal illumination images

Images of the posterior surface of the cornea seen in retro-illumination were obtained using a modified slit-lamp microscope. The light source of the slit-lamp was replaced with an LED emitting at 780 nm (THORLABS, Newton, NJ USA—ref M780L3) and the microscope was equipped with a 12.3 megapixel monochrome camera (type VCXU-123 M, BAUMER SAS, Fillinges, France). Images were obtained from patients with FECD and from the non-FECD control subjects under the framework of a clinical trial validated by the French National Agency for the Safety of Medicines and Health Products and an ethics committee (ID-RBC 2021-A01496-35). Representative images were shown.

### Acquisition of DM samples

DM samples approximately 8 mm diameter were obtained from 10 patients with FECD who underwent descemetorhexis during endothelial keratoplasty performed at the University Hospital Center of Saint Etienne. Slit-lamp microscopy revealed corneal edema in all patients with FECD. These DMs are normally discarded as the standard procedure during endothelial keratoplasty. Control DMs were obtained from 10 donor corneas that had been authorized for scientific use by the French Biomedical Agency (PFS15-008). The average age of the patients with FECD was 69 ± 14 (44, 90) years old, and the average age of the non-FECD donors was 78 ± 13 (52, 104) years old (*P* = 0.16). DMs were obtained from 4 males and 6 females in both the non-FECD and FECD groups. In the non-FECD group, the average post mortem time was 18.2 ± 6.3 (7, 24) hours (Table 1). All FECD and non-FECD subjects were residents of France. The DMs of the patients with FECD were preserved in CorneaMax (Eurobio, France) during the surgery, and transferred to the laboratory for processing for further experiments within 1 h. Control donor corneas were preserved in CorneaMax, and the absence of guttae was confirmed by microscopy. An approximately 11–12 mm diameter segment of each DM was peeled using the standardized “no-touch” technique^42^.

### Shotgun proteomics

Peeled DMs were gently rinsed with BSS™ Sterile Irrigating Solution (Alcon Laboratories, Inc., Geneva, Switzerland) and spread on a glass slide with the endothelial surface upward. This process completely removed the corneal endothelial cells. The DMs (Samples ID C7 and F7 shown in Table 1) were then cut into two pieces, and one piece was used for shotgun proteomics and the fellow piece for MALDI-IMS. The DMs were dried at room temperature and stored at − 80 °C until used for the experiment. Peptides were extracted from the tissue samples in 25–50 μL of 0.1% trifluoroacetic acid (TFA), stored at − 80 °C, and analyzed by LC–MS/MS (timsTOF Pro; Bruker, Billerica, MA) with nanoElute (Bruker). Mass spectra obtained by LC–MS/MS were analyzed by ProteinScape (Bruker), and the peptides and proteins were identified. Samples ID C1, C2, F1, and F2 (Table 1) were used for preliminary experiments to confirm that shotgun proteomics is feasible in flat-mounted DMs.

### Enrichment analyses

The Database for Annotation, Visualization, and Integrated Discovery (DAVID; http://david.ncifcrf.gov) was utilized to conduct functional enrichment analyses^43^. Gene Ontology (GO) analysis was performed to investigate the functions of the proteins identified by shotgun proteomics. The GO terms consisted of 3 categories: biological process (BP), cellular component (CC), and molecular function (MF). Reactome pathway analysis was also conducted using DAVID. Significantly enriched GO terms and pathways were considered with the threshold of *P*-value < 0.05, and the top-ranked GO and pathways were visualized as graphs generated in R with the “ggplot2” package.

### Immunofluorescence staining

The immunofluorescence staining of each protein was repeated for DMs from non-FECD subjects (77, 104, and 76 years old) (Sample ID C8-10 shown in Table 1) and from patients with FECD (58, 77, and 90 years old) (Sample ID F8-10 shown in Table 1). The DMs were cut into several pieces and treated with multiple antibodies to spare the samples. The immunostaining protocol was previously developed and validated for flat-mounted whole corneas^44,45^. Briefly, the samples were rehydrated in phosphate buffered saline (PBS) at room temperature for 5 min, followed by permeabilization with 0.5% Triton 100-X (EuroMedex, Souffelweyersheim, France). Non-specific binding sites were blocked by incubation in the blocking buffer (PBS supplemented with 2% heat-inactivated goat serum and 2% bovine serum albumin) for 30 min at 37 °C. The following primary antibodies were used after dilution in blocking buffer at 1/500: fibrinogen α (Abcam, ab34269), hemoglobin α (Santa Cruz Biothechnology, sc-514378), hemoglobin γδεβ (Santa Cruz Biothechnology, sc-390668), SRPX2 (Abcam, ab91584), and tenascin-C (Abcam, ab3970). The secondary antibodies, Alexa Fluor 488 goat anti-mouse (A32723, Invitrogen) or Alexa Fluor 555 goat anti-rabbit (A32732, Invitrogen), were diluted in blocking buffer at 1/1000 dilution. The samples were incubated for 60 min at 37 °C in each solution. Nuclei were counterstained with 2 µg/mL DAPI (D1306, Invitrogen) in PBS at room temperature for 10 min. The samples were given 3 rinses with PBS between all steps, except between the saturation step for non-specific protein binding sites and incubation with primary antibody. The flat mounts were covered with fluorescence mounting medium (NB-23-00158-2, Neo Biotech, Nanterre, France) and a glass coverslip. Images were acquired using an epifluorescence inverted microscope IX81 (Olympus, Tokyo, Japan) equipped with cellSens imaging software (cellSens Dimension, Olympus, Germany). The specificity of the markers was confirmed using non-specific rabbit and/or mouse IgG (Zymed, Carlsbad, CA) as primary antibodies for negative controls. The secondary antibodies for the controls were the same as those for the targeted proteins.

### MALDI-IMS

For protein imaging, flat-mounted DMs (Samples ID C7 and F7 shown in Table 1) were washed with 70 to 100% ethanol and then sprayed with 10 mg/mL a-cyano-4-hydroxycinnamic acid in 70% acetonitrile containing 1% TFA using an automated sprayer (TM-Sprayer; HTX technologies, Chapel Hill, NC). Mass spectra were measured using a Rapiflex Tissuetyper (Bruker) with a spatial resolution of 50 μL. The DMs were then sprayed with trypsin solution (25 mg/mL in 20 mM aqueous NH_4_HCO_3_, pH 7.5–8.5) at room temperature and incubated for 2 h at 50 °C. MALDI-IMS data were obtained and analyzed using flexImaging 5.0 and SCiLS Lab 2018b (Bruker). For protein analysis, PEAKS Studio 8.5 (Bioinformatics Solutions Inc., Ontario, Canada), ProteinScape (Bruker), and MASCOT software (Matrix Science, London, UK) were employed. Samples ID C3-6 and F3-6 (Table 1) were used for preliminary experiments to confirm that MALDI-IMS is feasible in flat-mounted DMs.

## Supplementary Information

Supplementary Figure 1.

## Data Availability

The datasets generated and analyzed during the current study are available in the Mass Spectrometry Interactive Virtual Environment (MassIVE; https://massive.ucsd.edu/ProteoSAFe/static/massive.jsp) repository with the accession ID: MSV000091078.
