# Supplementary material for: Shotgun proteomics identification of proteins expressed in the Descemet’s membrane of patients with Fuchs endothelial corneal dystrophy
Source: Sci Rep. 2023 Jun 27;13:10401. doi: 10.1038/s41598-023-37104-1 (PMC10300001; doi:10.1038/s41598-023-37104-1)
Supplement: Supplementary file 1 — Supplementary Figure 1. [file 41598_2023_37104_MOESM1_ESM.pdf]

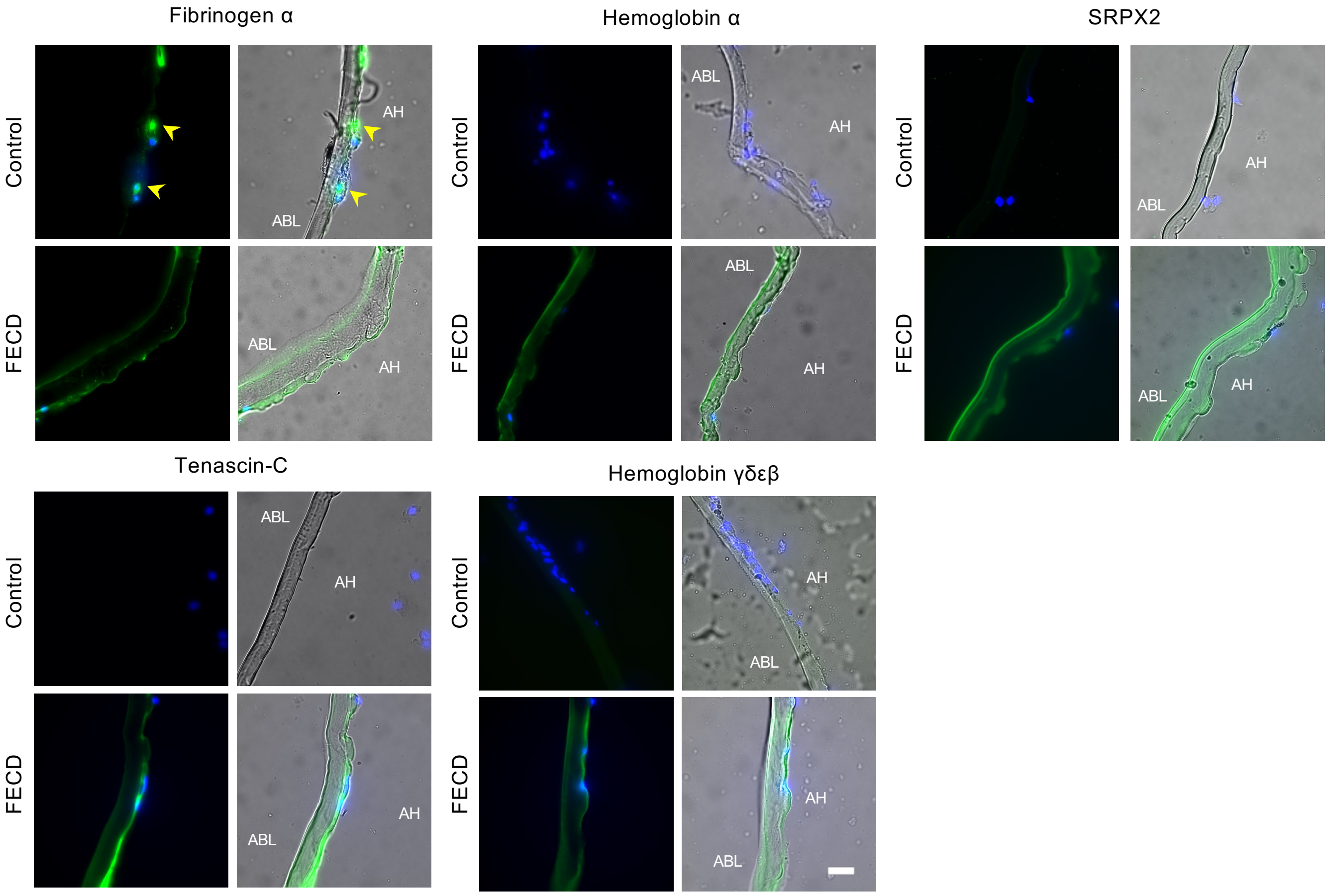

Supplementary figure 1. Immunostaining of cross-sections of Descemet's membrane (DM) of subjects with and without FECD (Fuchs endothelial corneal dystrophy)

The expression of the top 5 of 32 proteins expressed only in the FECD-DM but not in the non-FECD-DM was validated by immunofluorescence staining of frozen DM cross-sections. The images were observed using an epifluorescence microscope with a ×60 objective. In the non-FECD control samples, only fibrinogen α showed positive staining, whereas none of the top 5 proteins (fibrinogen α, hemoglobin α, SRPX2, tenascin-C, and hemoglobin γδεβ) were detected. Yellow arrows indicate perinuclear expression of fibrinogen α in non-FECD-DM. In the FECD-DM, fibrinogen α, hemoglobin α, SRPX2, tenascin-C, and hemoglobin γδεβ were clearly detected by immunostaining. The nuclei were counterstained with DAPI. The right columns show the immunostaining image combined with bright-field imaging. AH indicates the aqueous humor (endothelial cell side) and ABL indicates an anterior banded layer (stromal side of DM). Immunostaining of each protein was repeated for 3 non-FECD-DMs and 3 FECD-DMs. The scale bar: 20 μm.
